# High‐Dose Inhaled Colistin–Aminoglycoside Combination Therapy in Critically Ill Patients With Multidrug‐Resistant *Pseudomonas aeruginosa* Hospital/Ventilator‐Associated Pneumonia: A Case Report and Literature Review

**DOI:** 10.1155/crcc/9472232

**Published:** 2026-09-27

**Authors:** Thang Nguyen Ngoc, Cuong Duong Cao, Thang Vu, Soan Hoang, Quyet Nguyen

**Affiliations:** ^1^ Emergency Center, Phenikaa University Hospital, Hanoi, Vietnam; ^2^ Outpatient Department, Vinmec International Hospital, Hanoi, Vietnam; ^3^ College Of Medicine, Phenikaa University, Hanoi, Vietnam, phenikaa-uni.edu

## Abstract

**Background:**

Multidrug‐resistant *Pseudomonas aeruginosa* (MDR PA) ventilator‐associated pneumonia (VAP) and hospital‐associated pneumonia (HAP) are associated with high morbidity and limited therapeutic options. Intravenous antibiotics often achieve inadequate pulmonary concentrations. Aerosolized delivery permits high local drug exposure, but data on combined inhaled polymyxin–aminoglycoside therapy remain limited.

**Case Report:**

We describe three critically ill patients with HAP/VAP due to MDR PA. All received adjunctive high‐dose inhaled colistin immediately followed by inhaled amikacin or tobramycin, in addition to systemic therapy. Dosing was individualized based on renal function. Nebulization was optimized using inspiratory‐synchronized delivery. All patients demonstrated clinical improvement, reductions in inflammatory markers (though with a transient rise in one or more markers in two patients), radiologic resolution, and eventual microbiologic eradication, despite isolates reported as resistant to the aminoglycoside and colistin used in vitro. No nephrotoxicity, bronchospasm, or device‐related complications were observed clinically, despite doses exceeding those commonly reported, although formal ototoxicity surveillance was not performed. However, because all patients also received concurrent systemic antibiotics, a specific causal contribution from inhaled therapy cannot be fully established.

**Conclusions:**

Dual inhaled colistin–aminoglycoside therapy may represent a pharmacokinetically rational adjunctive strategy in selected patients with MDR PA HAP/VAP. High intrapulmonary concentrations achieved through aerosolization may overcome resistance while maintaining acceptable systemic safety. Prospective PK‐guided studies are warranted.

## 1. Introduction

Ventilator‐associated pneumonia (VAP) and hospital‐associated pneumonia (HAP) caused by *Pseudomonas aeruginosa* (PA) remain among the most challenging infectious complications in critical care. The rapid emergence of multidrug‐resistant (MDR) and extensively drug‐resistant (XDR) strains has severely limited therapeutic options, frequently leaving clinicians dependent on agents that either achieve suboptimal epithelial lining fluid (ELF) concentrations or are associated with significant systemic toxicity.

Although intravenous (IV) antibiotics—including carbapenems, polymyxins, and fluoroquinolones—remain standard therapy, pulmonary penetration is variable and often inadequate in mechanically ventilated patients. These pharmacokinetic limitations have renewed interest in aerosolized antibiotic delivery, which bypasses the alveolar–capillary barrier and enables markedly higher local drug concentrations while minimizing systemic exposure.

Among inhaled agents, colistin and aminoglycosides (amikacin or tobramycin) have individually demonstrated potential benefit as adjunctive therapy in MDR gram‐negative pneumonia. However, randomized studies have shown inconsistent clinical outcome benefits despite improved microbiologic eradication, and data regarding combined inhaled therapy remain scarce. A strong pharmacodynamic rationale supports dual therapy: polymyxins disrupt the gram‐negative outer membrane, thereby enhancing aminoglycoside intracellular penetration, lowering effective MICs, and accelerating bactericidal activity. This synergy may be particularly relevant in isolates categorized as resistant in vitro but potentially suppressible with very high local pulmonary concentrations.

We report three critically ill patients with HAP/VAP due to MDR PA. Each received adjunctive dual inhaled therapy with colistin followed immediately by amikacin or tobramycin, in addition to systemic antibiotics. All demonstrated rapid clinical and radiologic improvement and microbiologic clearance without significant adverse effects. These cases highlight a lung‐targeted, pharmacokinetically rational adjunctive strategy that warrants further prospective evaluation.

## 2. Method

### 2.1. Nebulization Technique

In all cases, inhaled antibiotics were administered using an inspiratory‐phase–synchronized system with a Hamilton C3 ventilator. A vibrating‐mesh nebulizer was used in Cases 1 and 3 and a jet nebulizer in Case 2. The nebulizer was positioned on the inspiratory limb approximately 10–15 cm from the Y‐piece. During nebulization, volume‐controlled ventilation (VCV) was used, and humidification was temporarily discontinued to optimize distal drug deposition. Patients were maintained at a RASS of −2 to −3 to ensure adequate patient–ventilator synchrony and minimize drug‐delivery variability.

Colistin was administered first, immediately followed by the aminoglycoside, to potentially enhance aminoglycoside penetration through membrane permeabilization. Each session lasted up to 60 min or until the nebulizer chamber was visibly empty, whichever occurred first. The treatment duration for each case is detailed in Table 1.

**Table 1:** Serial clinical, laboratory, respiratory, microbiological, and antimicrobial treatment findings in three patients during the course of treatment.

	Variable	Day 1	Day 2	Day 3	Day 4	Day 5	Day 6	Day 8	Day 11	Day 15
Case 1 75‐year‐old man with distal esophageal carcinoma underwent open gastrostomy	CPIS	11	NA				9	NA		
	SOFA	6	NA	7	NA	6	NA			
	Maximum body temperature (°C)	38.5	39	39	38.6	38.4	38.4	37.4	36.6	36.6
	Body weight (kg)	57	NA			56	NA			57
	WBC (G/L)	29.92	NA	18.51	NA	19.02	19.18	20.15	13.37	7.93
	NEU (G/L)	26.58	NA	15.79	NA	17.34	17.11	17.46	11.88	6.83
	Tracheal secretions	Copious purulent				Purulent	Purulent	Less Purulent	Clear	No Secretion
	Oxygenation (PaO_2_/FiO_2_)	170	160	140	150	160	160	240	290	NA
	Respiratory status	MV							NC	SB
	Procalcitonin (ng/mL)	1.95	NA	1.03	NA	0.195	NA	NA	NA	0.124
	BUN (mg/dL)	22	23	22	NA	20	NA	21	18	15
	Creatinine (mg/dL)	1.6	2	1.7	NA	1.7	NA	1.6	1.5	1
	Quantitative ETA culture (CFU/mL)	MDR PA (3 × 10^6^)	NA					MDR PA (< 10^4^) CA (2 × 10^4^)	MDR PA (< 10^4^)	(−)
	Blood Culture (from two different positions)	Negative	NA	Negative	NA	NA	NA	Negative	NA	NA
	Systemic antimicrobial therapy	Meropenem + vancomycin + moxifloxacin						Meropenem+ moxifloxacin		
	Inhaled colistin + aminoglycoside administered	No			Yes					No

Case 2	CPIS	10	NA				8	NA		
	SOFA	10	11	11	11	NA				
	Maximum body temperature (°C)	38.6	38.8	38.8	39	38.5	38,5	36.7	36.6	36.7
	Body weight (kg)	76.7	74.6	73.4	71	70	71	66	NA	62
	WBC (G/L)	16.04	NA	20.11	NA	9.9	11.41	8.94	NA	6.67
	NEU (G/L)	12.54	NA	17.82	NA	7.53	8.65	6.1	NA	4.32
	Tracheal secretions	Abundant purulent				Purulent	Purulent	Less purulent	Clear	No secretion
	Respiratory status	MV						NIV	NIV	SB
	Oxygenation (PaO_2_/FiO_2_)	140	150	130	110	110	110	210	300	NA
	Procalcitonin (ng/mL)	1.96	NA	0.96	NA	0.70	NA	0.63	NA	0.50
	BUN (mg/dL)	55	44	37	NA	33	NA	25	28	31
	Creatinine (mg/dL)	9.2	8.8	6.4	NA	5.9	NA	3.8	3.2	3.6
	Quantitative ETA culture (CFU/mL)	MDR PA (8 × 10^7^)	NA			MDR PA (5 × 10^7^)	NA	Negative	NA	Negative
	Blood culture (from two different positions)	MDR PA (2/2)	NA			Negative	NA	Negative	NA	Negative
	Systemic antimicrobial therapy	Colistin + meropenem + vancomycin + TMP‐SMX + fluconazole							Colistin + meropenem + TMP‐SMX	TMP‐SMX
	Inhaled colistin + aminoglycoside administered	No			Yes					No

Case 3	CPIS	5	NA							
	SOFA	8	8	9	NA	NA	NA	NA	NA	NA
	Maximum body temperature (°C)	35.9	38	38.4	38	38.6	38	36.3	36	35.7
	Body weight (kg)	55.6	NA	56	54.5	NA				54
	WBC (G/L)	22.65	NA	15.19	NA	13.88	14.34	NA	10.5	9.28
	NEU (G/L)	20.26	NA	10.93	NA	11.40	12.23	NA	7.5	5.82
	Tracheal secretions	Mildly Purulent	Copious purulent	Copious purulent	Purulent	Purulent	Purulent	Less purulent	Less purulent	Clear
	Respiratory status	MV						NIV	NIV	SB
	Oxygenation (PaO_2_/FiO_2_)	180	170	155	200	160	160	250	280	NA
	Procalcitonin (ng/mL)	0.345	NA	0.233	NA	1.06	NA	0.151	0.067	NA
	BUN (mg/dL)	15	NA	16	NA	13	11	NA	NA	11
	Creatinine (mg/dL)	1.1	NA	1.6	NA	0.9	1	NA	NA	0.9
	Quantitative ETA culture (CFU/mL)	Negative	NA	MDR PA (8 × 10^5^) CA (5 × 10^4^)	NA	MDR PA (8 × 10^5^)	NA	NA	MDR PA (< 10^4^)	Negative
	Blood Culture (from two different positions)	Negative	NA	Negative	NA	Negative	NA	NA	Negative	NA
	Systemic antimicrobial therapy	Levofloxacin + piperacillin/tazobactam					Levofloxacin + ceftazidime			NA
	Inhaled colistin + aminoglycoside administered	No				Yes				No

Abbreviations: BUN, blood urea nitrogen; CA, *Candida albicans*; CFU, colony‐forming units; ETA, endotracheal aspirate; ET, endotracheal; MDR, multidrug‐resistant; MDR PA, multidrug‐resistant *Pseudomonas aeruginosa*; MV, mechanical ventilation; NA, not available/not assessed; NC, nasal cannula; NEU, neutrophil count; NIV, noninvasive ventilation; PaO_2_/FiO_2_, ratio of arterial oxygen tension to inspired oxygen fraction; SB, spontaneous breathing; SOFA, Sequential Organ Failure Assessment; WBC, white blood cell count.

Patients were closely monitored during nebulization for any adverse effects or clinical deterioration. Serial renal function and clinical assessments for potential drug‐related toxicity were performed throughout treatment. Therapy was discontinued or modified if evidence of systemic accumulation or toxicity emerged.

### 2.2. Diagnostic Criteria for HAP/VAP, Treatment‐Failure Definition and Ethics

HAP was defined as pneumonia developing ≥ 48 h after hospital admission and not present or incubating at the time of admission. VAP was defined as pneumonia developing > 48 h after initiation of mechanical ventilation (MV). Diagnosis was based on the presence of a new or progressive pulmonary infiltrate on chest radiography or CT together with clinical evidence of infection, including fever or hypothermia, leukocytosis or leukopenia, purulent tracheal secretions, and/or worsening oxygenation or ventilatory requirements. Microbiological evidence, including isolation of a compatible pathogen from respiratory specimens, was used to support the diagnosis but was not considered diagnostic in isolation. Alternative explanations for the clinical and radiological findings were considered and excluded where clinically appropriate.

Failure of systemic antibiotic therapy, prompting initiation of adjunctive inhaled therapy, was defined as persistence or worsening of clinical, physiological, or microbiological evidence of infection despite 3–4 days of empiric or susceptibility‐guided IV antibiotic therapy. This included persistent fever, persistent or worsening purulent tracheal secretions and/or bilateral crackles, worsening oxygenation (PaO_2_/FiO_2_ ratio) or increasing ventilatory support requirements, persistent microbiological positivity, and/or failure of procalcitonin (PCT) to improve. Alternative clinical diagnoses or explanations for the lack of improvement were considered and excluded where appropriate before escalation to inhaled therapy; the day‐by‐day clinical, laboratory, and antimicrobial data supporting this determination for each case are presented in Table 1.

The use of adjunctive high‐dose inhaled therapy in each case followed multidisciplinary discussion among the treating intensivists and a clinical pharmacist, given the absence of an on‐site clinical microbiologist, documented failure of guideline‐concordant systemic therapy, and lack of any remaining evidence‐based systemic option. Written informed consent for treatment and for publication of patient data was obtained from the patients′ next of kin/legal representatives, consistent with institutional policy at Phenikaa University Hospital.

## 3. Case Presentation

### 3.1. Case 1

A 75‐year‐old man with distal esophageal carcinoma underwent open gastrostomy. His postoperative recovery was uneventful. Fourteen days later, he developed fever, copious purulent secretions, and acute respiratory failure requiring ICU admission and MV.

On ICU Day 1, CPIS was elevated at 11, and PCT measured 1.95 ng/mL. Chest radiography showed new bilateral infiltrates (Figure 1A). After other potential causes of postoperative infection were excluded, empiric IV meropenem, vancomycin, and moxifloxacin were initiated for suspected HAP.

**Figure 1:**
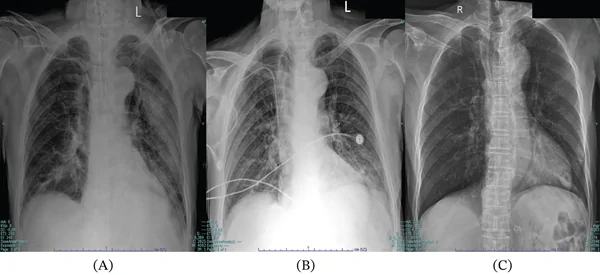
(A) Chest radiograph on ICU admission showing new, extensive bilateral patchy and confluent infiltrates, more pronounced in the left mid‐to‐lower lung zones, consistent with severe pneumonia. Endotracheal and central venous catheters are appropriately positioned. (B) Radiograph obtained on ICU Day 6, demonstrating marked interval improvement with decreasing bilateral infiltrates and improved lung aeration. (C) Follow‐up radiograph at Day 17 after completion of therapy showing near‐complete resolution of previous infiltrates and normalization of lung fields.

Endotracheal aspirate cultures (EAC) at ICU Day 1 grew MDR PA resistant to all routinely available IV agents (Table 2). Due to lack of clinical response, adjunctive inhaled therapy was initiated:

• Colistin 2 MIU every 12 h.
• Immediately followed by amikacin 1 g every 12 h.

**Table 2:** Antimicrobial susceptibility profiles of multidrug‐resistant *Pseudomonas aeruginosa* isolated from endotracheal aspirate cultures in three cases. MIC, minimum inhibitory concentration; R, resistant. MIC values are expressed in *μ*g/mL. Susceptibility interpretations were based on Clinical and Laboratory Standards Institute (CLSI) M100, 32nd edition (2022) according to the laboratory′s testing standards.

Antimicrobial agent	Case 1	Case 2	Case 3
Levofloxacin (*μ*g/mL)	8 (R)	16 (R)	16 (R)
Ciprofloxacin (*μ*g/mL)	4 (R)	8 (R)	8 (R)
Amikacin (*μ*g/mL)	64 (R)	64 (R)	64 (R)
Tobramycin (*μ*g/mL)	8 (I)	16 (R)	16 (R)
Ceftazidime (*μ*g/mL)	32 (R)	64 (R)	16 (I)
Imipenem (*μ*g/mL)	≥ 16 (R)	≥ 16 (R)	≥ 16 (R)
Meropenem (*μ*g/mL)	≥ 16 (R)	≥ 16 (R)	≥ 16 (R)
Piperacillin (*μ*g/mL)	≥ 128 (R)	≥ 128 (R)	64 (I)
Colistin (*μ*g/mL)	8 (R)	4 (R)	4 (R)

Both agents were diluted in normal saline and delivered via vibrating‐mesh nebulizer.

The patient′s clinical course was biphasic. After initiation of inhaled therapy, secretion burden and oxygenation improved, and PCT fell progressively (1.95 → 0.124 ng/mL). WBC, however, followed a nonmonotonic trajectory, transiently rising from 18.51 G/L (Day 3) to a peak of 20.15 G/L (Day 8) before declining to 7.93 G/L by Day 15 (Table 1). EAC remained intermittently positive for MDR PA through Day 11 and converted to negative only by Day 15, with transient coisolation of *Candida albicans* (CA) on Day 8. Serial imaging showed near‐complete radiologic resolution (Figure 1B,C).

He was extubated on ICU Day 10. Sputum became sterile by Day 15. No nephrotoxicity, bronchospasm, or neurotoxicity occurred. The patient was subsequently discharged to the Cancer Center to resume his cancer treatment.

### 3.2. Case 2

A 63‐year‐old woman with a recent prolonged ICU stay following intracerebral hemorrhage was transferred with metabolic acidosis, acute kidney injury requiring CRRT, and suspected VAP. On admission, she had abundant purulent tracheal secretions and bilateral pulmonary infiltrates on chest radiography (Figure 2A). Given marked fluid overload, daily negative fluid balance was pursued, resulting in a steady decline in body weight. Empiric IV meropenem, vancomycin, trimethoprim‐sulfamethoxazole, and fluconazole were initiated on ICU Day 1. Despite these measures, patient′s clinical findings continued to deteriorate; adjunctive inhaled therapy was added on Day 4 with:

• Colistin 4 MIU every 12 h.
• Immediately followed by tobramycin 200 mg every 8 h.

**Figure 2:**
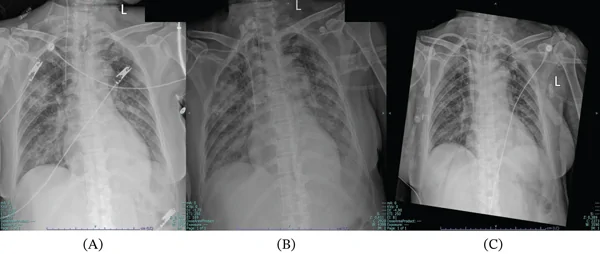
(A) Chest radiograph at admission showing diffuse bilateral interstitial and alveolar opacities with increased vascular markings. (B) Interval radiograph after a few days of systemic and adjunctive inhaled tobramycin–colistin therapy demonstrating partial clearing of infiltrates and improved aeration of both lungs. (C) Follow‐up radiograph at Day 12 showing marked radiologic improvement with substantial resolution of bilateral infiltrates, compatible with favorable therapeutic response.

Nebulization was delivered via a jet nebulizer. IV colistin was coadministered due to bacteremia risk. Endotracheal aspirate and blood cultures obtained on ICU Day 1 confirmed MDR PA, resistant to available IV treatment options (Table 2). PCT declined progressively from 1.96 to 0.50 ng/mL. WBC followed a nonmonotonic course: it rose from 16.04 G/L at baseline to a peak of 20.11 G/L on Day 3, fell to 9.9 G/L by Day 5, rebounded slightly to 11.41 G/L on Day 6, and then declined to 6.67 G/L by Day 15 (Table 1). This was accompanied by marked radiological improvement (Figure 2B,C) and microbiological eradication by ICU Day 6, sustained through Day 15 (Table 1).

She was later weaned from CRRT and MV without adverse effects related to inhaled therapy. The patient was later discharged from the ICU and transferred to the rehabilitation center for her post‐stroke care and was subsequently discharged from the hospital with significant neurological and renal function recovery.

### 3.3. Case 3

A 90‐year‐old woman from a long‐term care facility presented with cardiogenic shock due to right ventricular STEMI, requiring intubation and PCI. Bilateral infiltrates were initially attributed to cardiogenic pulmonary edema and possible peri‐intubation aspiration in the setting of shock. However, despite adequate diuresis and hemodynamic stabilization, on ICU Day 2, she developed new fever and worsening of the bilateral crackles, together with purulent tracheal secretions, leukocytosis, and worsening oxygenation (Table 1), which was suggestive of VAP. EAC on Day 3 grew MDR PA resistant (Table 2). Adjunctive inhaled therapy was initiated on ICU Day 5:

• Colistin 4 MIU every 12 h.
• Immediately followed by amikacin 2 g every 12 h.

The patient showed steady clinical improvement despite a biphasic laboratory trajectory. PCT levels decreased from 0.345 to 0.233 ng/mL by ICU Day 3, followed by a transient rise to 1.06 ng/mL on Day 5 and then a progressive decline to 0.067 ng/mL by Day 11 (Table 1). The WBC count similarly decreased from 22.7 to 9.3 G/L. CT imaging demonstrated near‐complete resolution of the bilateral pulmonary consolidations (Figures 3)A,B. However, EACs remained intermittently positive for MDR PA through Day 11, with transient coisolation of *CA* on Day 3; microbiological eradication of *PA* was ultimately achieved by ICU Day 15 (Table 1).

**Figure 3:**
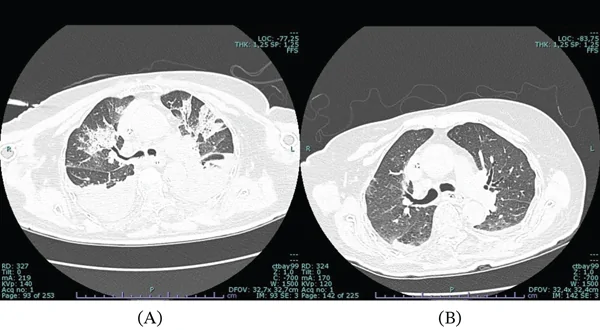
(A) A computed tomographic (CT) section on Day 1 (before antibiotic administration) at the carina level, with lung regions characterized by extensive bilateral patchy consolidations and ground‐glass opacities, more pronounced in the lower lobes and peribronchial regions, suggestive of active infectious or inflammatory pneumonia. (B) The corresponding CT section (according to pulmonary vessels, bronchi, and mediastinal structures) on Day 10; there is a notable reduction in the extent and density of consolidations, with clearer aeration of both lungs and decreased inflammatory opacities. Residual linear changes and mild ground‐glass areas persist, suggesting resolving pneumonia or postinfectious parenchymal healing.

No bronchospasm, nephrotoxicity, neurotoxicity, or ventilator circuit complications occurred. She was successfully extubated and discharged in stable condition into her nursing care.

## 4. Discussion

The therapeutic rationale for inhaled antibiotics was first established in chronic airway infections. In cystic fibrosis, persistent *PA* colonization drives progressive structural damage, and randomized trials demonstrated that inhaled tobramycin and colistin reduce bacterial density, improve lung function, and prolong time to exacerbation [1, 2]. Similar benefits were confirmed in noncystic fibrosis bronchiectasis [3]. These findings provided the pharmacologic and translational foundation for aerosolized therapy in acute critical illness, where achieving adequate intrapulmonary exposure with IV therapy alone is often difficult. The global rise of MDR gram‐negative pathogens, particularly *PA*, poses major therapeutic challenges in the ICU. VAP remains associated with prolonged hospitalization, mortality, and substantial cost [4]. In this context, alternative delivery strategies aimed at maximizing pulmonary drug exposure are increasingly relevant.

A notable observation in our series is that clinical improvement occurred alongside inhaled aminoglycoside and colistin therapy despite in vitro resistance to these agents in at least one isolate (Table 2). Given the concurrent systemic therapy and other confounders, we present this as a hypothesis‐generating observation rather than evidence of a causal resistance‐overcoming effect. One plausible biological explanation lies in the pharmacokinetic advantages of aerosolized delivery. Nebulization bypasses the alveolar–capillary barrier and achieves ELF concentrations several orders of magnitude higher than IV administration. Experimental pneumonia models demonstrate lung tissue amikacin concentrations up to 30‐fold higher with nebulization [5]. Large‐animal microdialysis studies confirm markedly higher interstitial and ELF concentrations after inhaled versus IV aminoglycosides, with comparable systemic exposure [6, 7]. Early ELF concentrations exceed interstitial levels by orders of magnitude, underscoring preferential airway targeting.

For aminoglycosides, nebulized administration can achieve systemic exposure comparable with IV dosing when accounting for extrapulmonary deposition, while simultaneously generating markedly higher drug concentrations within the airways [6]. Systemic absorption may still occur, particularly when alveolar–capillary barrier integrity is compromised, with peak plasma levels observed around 1 h and trough concentrations similar to those achieved intravenously. Notably, nebulized tobramycin has been shown to produce greater lung interstitial and ELF concentrations than IV administration, without increasing systemic exposure [7]. Similarly, inhaled colistin achieves ELF concentrations several‐fold higher than plasma levels [8, 9]. Higher doses can produce ELF concentrations exceeding 1000 mg/L—far above systemic exposure [10]. Systemic diffusion remains limited even in extensive VAP [5, 8–10], permitting delivery of very high local concentrations with reduced nephrotoxicity risk. However, standard colistin regimens (e.g., 80 mg every 8 h) may not sustain ELF concentrations above contemporary EUCAST breakpoints throughout the dosing interval, particularly for resistant pathogens, supporting individualized higher dose strategies [9, 11].

Drug deposition within the injured lung is heterogeneous. Experimental colistin pneumonia models show greater tissue concentrations in mildly infected compared with consolidated segments, with superior bacterial killing after nebulized versus IV therapy [6]. Extrapulmonary deposition depends on ventilator configuration, humidification, device performance, inspiratory flow, and lung mechanics [12]. Although scintigraphy, circuit mass‐balance analysis, and ELF sampling can estimate deposition under experimental conditions, no validated bedside method precisely quantifies delivered lung dose in mechanically ventilated ICU patients. Consequently, dosing in critical illness remains partly empirical.

### 4.1. Individualized High‐Dose Strategy and Sequence Optimization

Guided by these pharmacokinetic principles, dosing was individualized based on renal function and illness severity:

• Patient 1: colistin 2 MIU q12 h → amikacin 1 g q12 h (mild renal impairment).
• Patient 2: colistin 4 MIU q12 h → tobramycin 200 mg q8 h (on CRRT).
• Patient 3: colistin 4 MIU q12 h → amikacin 2 g q12 h (normal renal function).

Colistin was deliberately administered first to disrupt the gram‐negative outer membrane and enhance aminoglycoside intracellular penetration, lowering effective MICs and accelerating bactericidal activity [13, 14]. This membrane‐permeabilizing effect may be particularly relevant in isolates categorized as aminoglycoside‐resistant in vitro. Experimental data further demonstrate that colistin–aminoglycoside combinations eradicate mature *PA* biofilms at lower concentrations than monotherapy and retain activity against resistant strains [13, 14].

Our doses exceeded those commonly reported in adult ICU series [8, 11, 15, 16]. Nevertheless, no nephrotoxicity or clinically significant adverse events occurred. Daily renal monitoring was performed, consistent with the systemic absorption profile of aminoglycosides. Ventilator optimization was rigorously applied to maximize distal drug deposition, similarly reported in other studies [17–19]. No bronchospasm, derecruitment, hypoxemia, or device‐related complications were observed. *CA* was transiently coisolated from endotracheal aspirates in Cases 1 and 3 (Days 8 and 3, respectively), which suggested airway colonization rather than invasive candidiasis. Neither patient received antifungal therapy, and subsequent cultures cleared spontaneously, supporting colonization rather than infection. Case 2 received empiric fluconazole as part of the initial broad‐spectrum regimen due to illness severity and prior history of prolonged ICU stay, despite no *CA* isolation from respiratory or blood cultures.

Randomized trials of adjunctive inhaled antibiotics in VAP demonstrate improved microbiologic eradication but inconsistent mortality benefit. This discrepancy likely reflects heterogeneity in patient selection, nonstandardized nebulization techniques, and variable device performance [16]. Systematic reviews emphasize the need for standardized delivery protocols and predefined PK/PD endpoints [19]. Current guidelines therefore restrict inhaled antibiotics to adjunctive use in MDR gram‐negative pneumonia not responding to systemic therapy [20–22]. Importantly, these recommendations are weak and reflect limited high‐quality evidence rather than definitive harm.

## 5. Limitations and Future Directions

This report has several limitations. Causality cannot be established definitively because all patients received concomitant systemic antibiotics; in Case 2, concurrent IV colistin further limits attribution of the response to inhaled therapy; the absence of quantitative or protected‐specimen BAL sampling, airway colonization cannot be completely excluded. ELF concentrations were not measured, precluding direct assessment of pulmonary drug exposure, and formal synergy testing of the colistin–aminoglycoside combination was not performed; the rationale for combination therapy was instead based on the mechanistic and experimental evidence discussed above. Serum amikacin and colistin therapeutic drug monitoring was not routinely performed, limiting detection of potential systemic accumulation. Inhaled antibiotic dosing remains nonstandardized, and the regimens used here may not be generalizable. Systematic audiological and vestibular monitoring was not performed, so the absence of reported ototoxicity reflects clinical observation rather than formal testing. Finally, this three‐patient uncontrolled case series is hypothesis‐generating rather than confirmatory.

Despite these constraints, our series highlights the underexplored strategy of dual inhaled colistin–aminoglycoside therapy for MDR PA HAP/VAP. The mechanistic pharmacokinetic rationale—supported by experimental and clinical data—suggests that high intrapulmonary concentrations may overcome conventional resistance thresholds while maintaining acceptable systemic safety. Prospective PK‐guided investigations and rigorously designed randomized trials using standardized nebulization protocols are required to define the role of dual inhaled therapy in modern critical care.

## Funding

No funding was received for this manuscript.

## Disclosure

All authors have read and approved the final version of the manuscript. The corresponding author had full access to all of the data in this study and takes complete responsibility for the integrity of the data and the accuracy of the data analysis. The corresponding author affirms that this manuscript is an honest, accurate, and transparent account of the study being reported; that no important aspects of the study have been omitted; and that any discrepancies from the study as planned have been explained.

## Conflicts of Interest

The authors declare no conflicts of interest.

## Data Availability

The data that support the findings of this study are available from the corresponding author upon reasonable request.

## References

[1] Ramsey B. W. , Pepe M. S. , Quan J. M. , Otto K. L. , Montgomery A. B. , Williams-Warren J. , Vasiljev-K M. , Borowitz D. , Bowman C. M. , Marshall B. C. , Marshall S. , and Smith A. L. , Intermittent Administration of Inhaled Tobramycin in Patients With Cystic Fibrosis, New England Journal of Medicine. (1999) 340, no. 1, 23–30, 10.1056/NEJM199901073400104, 9878641. 9878641

[2] Haworth C. S. , Foweraker J. E. , Wilkinson P. , Kenyon R. F. , and Bilton D. , Inhaled Colistin in Patients With Bronchiectasis and Chronic *Pseudomonas aeruginosa* Infection, American Journal of Respiratory and Critical Care Medicine. (2014) 189, no. 8, 975–982, 10.1164/rccm.201312-2208OC, 24625200. PMC4098097 24625200

[3] Amjad Z. , Abaza A. , Vasavada A. M. , Sadhu A. , Valencia C. , Fatima H. , Nwankwo I. , Anam M. , Maharjan S. , and Penumetcha S. S. , Inhaled Antibiotics in Non-Cystic Fibrosis Bronchiectasis (NCFB): A Systematic Review of Efficacy and Limitations in Adult Patients, Cureus. (2022) 14, no. 10, e30660, 10.7759/cureus.30660, 36439573. PMC9685586 36439573

[4] Papazian L. , Klompas M. , and Luyt C. E. , Ventilator-Associated Pneumonia in Adults: A Narrative Review, Intensive Care Medicine. (2020) 46, no. 5, 888–906, 10.1007/s00134-020-05980-0, 32157357. PMC7095206 32157357

[5] Rouby J. J. , Bouhemad B. , Monsel A. , Brisson H. , Arbelot C. , Lu Q. , and Nebulized Antibiotics Study Group , Aerosolized Antibiotics for Ventilator-Associated Pneumonia: Lessons From Experimental Studies, Anesthesiology. (2012) 117, no. 6, 1364–1380, 10.1097/ALN.0b013e3182755d7a. 23135264

[6] Rouby J. J. , Sole-Lleonart C. , Rello J. , Monsel A. , Constantin J. M. , and Bouglé A. , Ventilator-associated Pneumonia Caused by Multidrug-Resistant Gram-negative Bacteria: Understanding Nebulization of Aminoglycosides and Colistin, Intensive Care Medicine. (2020) 46, no. 4, 766–770, 10.1007/s00134-019-05890-w, 31915838. PMC7223812 31915838

[7] Dhanani J. A. , Diab S. , Chaudhary J. , Cohen J. , Parker S. L. , Wallis S. C. , Boidin C. , Barnett A. , Chew M. , Roberts J. A. , and Fraser J. F. , Lung Pharmacokinetics of Tobramycin by Intravenous and Nebulized Dosing in a Mechanically Ventilated Healthy Ovine Model, Anesthesiology. (2019) 131, no. 2, 344–355, 10.1097/ALN.0000000000002752, 31107274. 31107274

[8] Choi H. K. , Kim Y. K. , Kim H. Y. , and Uh Y. , Inhaled Colistin for Treatment of Pneumonia due to Colistin-Only-Susceptible *Acinetobacter baumannii* , Yonsei Medical Journal. (2014) 55, no. 1, 118–125, 10.3349/ymj.2014.55.1.118, 24339296. PMC3874897 24339296

[9] Athanassa Z. E. , Markantonis S. L. , Fousteri M. Z. F. , Myrianthefs P. M. , Boutzouka E. G. , Tsakris A. , and Baltopoulos G. J. , Pharmacokinetics of Inhaled Colistimethate Sodium (CMS) in Mechanically Ventilated Critically Ill Patients, Intensive Care Medicine. (2012) 38, no. 11, 1779–1786, 10.1007/s00134-012-2628-7, 22810779. 22810779

[10] Boisson M. , Jacobs M. , Grégoire N. , Gobin P. , Marchand S. , Couet W. , and Mimoz O. , Comparison of Intrapulmonary and Systemic Pharmacokinetics of Colistin Methanesulfonate (CMS) and Colistin after Aerosol Delivery and Intravenous Administration of CMS in Critically Ill Patients, Antimicrobial Agents and Chemotherapy. (2014) 58, no. 12, 7331–7339, 10.1128/aac.03510-14, 25267660. PMC4249558 25267660

[11] Lu Q. , Girardi C. , Zhang M. , Bouhemad B. , Louchahi K. , Petitjean O. , Wallet F. , Becquemin M. H. , le Naour G. , Marquette C. H. , and Rouby J. J. , Nebulized and Intravenous Colistin in Experimental Pneumonia Caused by *Pseudomonas aeruginosa* , Intensive Care Medicine. (2010) 36, no. 7, 1147–1155, 10.1007/s00134-010-1879-4, 20397007. 20397007

[12] Xu F. , He L. L. , Che L. Q. , Li W. , Ying S. M. , Chen Z. H. , and Shen H. H. , Aerosolized Antibiotics for Ventilator-Associated Pneumonia: A Pairwise and Bayesian Network Meta-Analysis, Critical Care. (2018) 22, no. 1, 10.1186/s13054-018-2106-x, 30442203. PMC6238320 30442203

[13] Wang Y. , Li C. , Wang J. , Bai N. , Zhang H. , Chi Y. , and Cai Y. , The Efficacy of Colistin Combined With Amikacin or Levofloxacin Against *Pseudomonas aeruginosa* Biofilm Infection, Microbiology Spectrum. (2022) 10, no. 5, e01468-22, 10.1128/spectrum.01468-22, 36102678. PMC9603716 36102678

[14] Ontong J. C. , Ozioma N. F. , Voravuthikunchai S. P. , and Chusri S. , Synergistic Antibacterial Effects of Colistin in Combination With Aminoglycoside, Carbapenems, Cephalosporins, Fluoroquinolones, Tetracyclines, Fosfomycin, and Piperacillin on Multidrug Resistant *Klebsiella pneumoniae* Isolates, PLoS One. (2021) 16, no. 1, e0244673, 10.1371/journal.pone.0244673, 33406110. PMC7787437 33406110

[15] Lu Q. , Yang J. , Liu Z. , Gutierrez C. , Aymard G. , Rouby J. J. , and Nebulized Antibiotics Study Group , Nebulized Ceftazidime and Amikacin in Ventilator-Associated Pneumonia Caused by *Pseudomonas aeruginosa* , American Journal of Respiratory and Critical Care Medicine. (2011) 184, no. 1, 106–115, 10.1164/rccm.201011-1894OC, 21474643. 21474643

[16] Kollef M. H. , Ricard J. D. , Roux D. , Francois B. , Ischaki E. , Rozgonyi Z. , Boulain T. , Ivanyi Z. , János G. , Garot D. , Koura F. , Zakynthinos E. , Dimopoulos G. , Torres A. , Danker W. , and Montgomery A. B. , A Randomized Trial of the Amikacin Fosfomycin Inhalation System for the Adjunctive Therapy of Gram-Negative Ventilator-Associated Pneumonia: IASIS Trial, Chest. (2017) 151, no. 6, 1239–1246, 10.1016/j.chest.2016.11.026, 27890714. 27890714

[17] Ari A. , Atalay O. T. , Harwood R. , Sheard M. M. , Aljamhan E. A. , and Fink J. B. , Influence of Nebulizer Type, Position, and Bias Flow on Aerosol Drug Delivery in Simulated Pediatric and Adult Lung Models During Mechanical Ventilation, Respiratory Care. (2010) 55, no. 7, 845–851, 10.4187/respcare.10550845, 20587095. 20587095

[18] O’Riordan T. G. , Palmer L. B. , and Smaldone G. C. , Aerosol Deposition in Mechanically Ventilated Patients. Optimizing Nebulizer Delivery, American Journal of Respiratory and Critical Care Medicine. (1994) 149, no. 1, 214–219, 10.1164/ajrccm.149.1.8111585. 8111585

[19] Sella N. , Pettenuzzo T. , De Cassai A. , Zarantonello F. , Congedi S. , Bruni A. , Garofalo E. , Ocagli H. , Gregori D. , Longhini F. , and Navalesi P. , Inhaled Antibiotics for Treating Pneumonia in Invasively Ventilated Patients in Intensive Care Unit: A Meta-Analysis of Randomized Clinical Trials With Trial Sequential Analysis, Critical Care. (2024) 28, no. 1, 10.1186/s13054-024-05159-9, 39587607. PMC11587605 39587607

[20] Torres A. , Niederman M. S. , Chastre J. , Ewig S. , Fernandez-Vandellos P. , Hanberger H. , Kollef M. , Li Bassi G. , Luna C. M. , Martin-Loeches I. , Paiva J. A. , Read R. C. , Rigau D. , Timsit J. F. , Welte T. , and Wunderink R. , International ERS/ESICM/ESCMID/ALAT Guidelines for the Management of Hospital-Acquired Pneumonia and Ventilator-Associated Pneumonia: Guidelines for the Management of Hospital-Acquired Pneumonia (HAP)/Ventilator-Associated Pneumonia (VAP) of the European Respiratory Society (ERS), European Society of Intensive Care Medicine (ESICM), European Society of Clinical Microbiology and Infectious Diseases (ESCMID) and Asociación Latinoamericana del Tórax (ALAT), European Respiratory Journal. (2017) 50, no. 3, 10.1183/13993003.00582-2017, 28890434. 28890434

[21] Kalil A. C. , Metersky M. L. , Klompas M. , Muscedere J. , Sweeney D. A. , Palmer L. B. , Napolitano L. M. , O′Grady N. P. , Bartlett J. G. , Carratalà J. , el Solh A. A. , Ewig S. , Fey P. D. , File T. M. , Restrepo M. I. , Roberts J. A. , Waterer G. W. , Cruse P. , Knight S. L. , and Brozek J. L. , Management of Adults With Hospital-Acquired and Ventilator-Associated Pneumonia: 2016 Clinical Practice Guidelines by the Infectious Diseases Society of America and the American Thoracic Society, Clinical Infectious Diseases. (2016) 63, no. 5, e61–e111, 10.1093/cid/ciw353, 27418577. PMC4981759 27418577

[22] Rello J. , Solé-Lleonart C. , Rouby J. J. , Chastre J. , Blot S. , Poulakou G. , Luyt C. E. , Riera J. , Palmer L. B. , Pereira J. M. , Felton T. , Dhanani J. , Bassetti M. , Welte T. , and Roberts J. A. , Use of Nebulized Antimicrobials for the Treatment of Respiratory Infections in Invasively Mechanically Ventilated Adults: A Position Paper From the European Society of Clinical Microbiology and Infectious Diseases, Clinical Microbiology and Infection. (2017) 23, no. 9, 629–639, 10.1016/j.cmi.2017.04.011, 28412382. 28412382

